# Sterilization of Male Insane*Chairman’s address before the Section on State Medicine and Public Hygiene of the State Medical Association of Texas, Galveston, May 11, 1909. F. E. Daniel, M. D., chairman.

**Published:** 1909-08

**Authors:** 


### EDITORIAL DEPARTMENT.

### Sterilization of Male Insane.*

The address of the chairman of a section is expected to be a learned disquisition on the matter pertaining to that section. Most addresses of the kind are. And, according to precedent, my paper on this occasion, if not learned, should at least set forth the many triumphs of sanitary science and "point with pride" to the wonderful advancements made in eradicating disease by the enforcement of its elementary principles. Therefore, I should be expected to recite the conquest of yellow fever, and other diseases, which formerly destroyed thousands; the regeneration of Cuba and Panama, the reduction of mortality from consumption under the systematic enforcement of cleanliness and ventilation; reform in tenement house construction, etc., and by contrast an address on State medicine should, by precedent, set forth in forcible language the necessity of further reforms. It should recite the fact that the Japs, a few generations removed from savagery, put us to the blush in life saving measures; and the further fact that during our brief brush with the Spaniards fourteen soldiers died of preventable disease in camp outside of Cuba for every one who fell in battle, according to Major L. L. Seaman, U. S. A.

But I realize that to follow precedent I should be a bore; that I would recite a thrice-told and threadbare tale. These things have been written and said until they cease to arrest attention, and I am going to strike out on a new line. I shall not follow precedent. Like Napoleon, who said, "I will be my own ancestor," I will make my own precedent, and I, therefore, beg your attention for not over ten minutes to a consideration of the advisability of sterilizing the males in all cases of incurable insanity. This is rather startling at first blush, but it is a maxim that "desperate cases require desperate remedies." The case in this instance is desperate. It calls for speedy relief; but the remedy proposed is not desperate. It is rational, and, as I shall show, has been used successfully in other States.

By way of exordium and a text for my argument I reproduce

---

*Chairman's address before the Section on State Medicine and Public Hygiene of the State Medical Association of Texas, Galveston, May 11, 1909. F. E. Daniel, M. D., chairman.

here some facts and figures from my paper on "Elements of Decay in American Civilization," which appeared in the *Texas State Journal of Medicine,* December, 1908. To these have been added the cost of the insane for the two years ending August 31, 1909:

"The population of Texas in forty-five years to 1904 increased 504 per cent. The insane in Texas in the same time increased 6800 per cent, a ratio of 13.7 to 1. In 1860 there were fifty insane people in Texas, and their maintenance cost the State $12,000. These figures are from a public and published address by Prof. M. L. Graves, M. D., University of Texas, late Superintendent Southwestern Insane Asylum, San Antonio, Texas.

"In 1909 there are 5000 insane in Texas (500 in jail for lack of asylum accommodation), and the cost to the State for the five years to 1904 was $3,555,000, an average of $707,000 a year. For 1904 alone the cost was $784,000. These figures are taken from the Comptroller's books.

"According to Dr. Graves, 60 per cent of the insane in Texas is the result of alcohol directly and by hereditary transmission; while Dr. B. M. Worsham, Superintendent of the Austin (Texas) Insane Asylum, puts it at 95 per cent."

In 1909 there are over 5000 insane in Texas, of which 10 to 20 per cent are in jails for want of accommodation, and the cost to the State for the two years ending August 31, 1909 (the three lunatic asylums and the epileptic colony), was $1,392,905. These figures are from the official record in the office of the Secretary of State, and are published in the official laws of Texas. In addition to this the Thirty-first Legislature has just appropriated $40,000 with which to found and put in operation a leprosy colony.

It is, indeed, truly astounding that we go on year after year away up in the opening decade of the Twentieth Century of the Christian era, dealing with effects and ignoring causes; spending millions of dollars of the people's money in the endeavor to care for the ever increasing number of victims of causes that should not operate or have ever operated. And yet we boast of our civilization. Are we civilized or even half enlightened? The Romans two thousands years ago put us of this age to shame in matters of public sanitation, especially with reference to sewers and architecture. They at least did not empty their sewers into their drinking water, as we do, nor, so far as I know, establish military camps on the watershed of their supply. I apprehend that a civilized people, in the sense that I understand civilization, would not permit the adulteration of everything they eat and drink, the sale of diseased meat and tuberculous milk, nor permit swarms of flies to scatter disease broadcast. It has been love's labor lost, or nearly lost, on the part of this Association in endeavoring to bring about reform, to correct the many evils of neglect of sanitation. It has been a difficult matter to interest those who make the laws in the subject of the public health, the one interest of paramount importance. In vain has it been pointed out by tongue and pen that the rational method of dealing with disease is to prevent it; that it is easier and cheaper to prevent than to cure it. In vain have been shown time and again the immense value of lives lost by preventable disease; and the economy—cost what it may—of sanitation. The "exceptional men" of the day have begged for the enactment of laws to enforce its principles. It is gratifying to state that after about twenty-five years of endeavor on the part of the Texas State Medical Association a public sentiment for reform has been awakened and protection of the public health was made a platform demand. In accordance with this demand the Thirty-first Legislature has given us a fairly good State Board of Health, though with very limited powers.* I must confine myself, however, to my text.

It will be seen by reference to the figures given above that insanity is increasing in Texas at a ratio out of all proportion to increase in population; and that it will continue to increase in the future as it has done in the past faster than provision can be made to care for the sufferers. The burden on the taxpayers of caring for them has already become appalling, approximating, as it does, three-quarters of a million dollars annually; while at least 10 per cent of the insane are not cared for; there is no room for them, and many of the indigent are in jail. What a commentary on our civilization! The four large State asylums and one large private asylum with an aggregate capacity of 5000, are crowded all the time, and the overflow must stay in jail until the State builds more accommodation. Where is this going to stop? It will not stop; for insanity, being a hereditary disease, is being transmitted to succeeding generations, and if conditions are not changed—if something is not not done to arrest it—it is a matter of simple calculation that in two more decades the insane will have increased until it will be, as it almost is now, practically impossible to care for

---

*P. S.—The Governor in his wisdom crippled it by cutting off nearly every help given the Board—appropriation and all—and is now being carricatured in the press, lay and medical, as standing in with the flies. Like Wm. Pitt, he has been called "the enemy of the human race."—(See *The State Journal of Medicine* for July.—D.)

them. I estimate that by 1930 Texas will require ten large asylums, at an annual cost of two and a half or three millions.

I have shown elsewhere (TEXAS MEDICAL JOURNAL) from statistics furnished by Dr. C. S. Gregory, Superintendent State Insane Asylum at Terrell, Texas, that in twenty-two asylums in sixteen States the cause of insanity in 46.75 per cent of the insane is alcohol, and a large part of this percentage is in the second and third generation of alcoholic insanity. This, of course, is only one cause of insanity, but it is the predominating cause. I have said that it is cheaper, easier and more rational to remove the cause than to deal with the effects. But it seems that the people are not yet civilized enough to appreciate the enormous and far-reaching ruinous effects of a policy that licenses the sale of an agent so destructive of health, home, fortune, happiness, life itself; a policy that multiplies crime and insanity, thus striking at the vitals of society, and undermining the integrity of the human family; that makes widows and orphans, murderers, thieves and paupers and prostitutes. The exceptional men of the race have not been able to convince the moneyed men who reap fortunes from and fatten on the sorrows of mankind that the saloon is not a good business proposition, in the face of the fact that in Texas the cost of caring for the victims of alcohol more than doubles the revenue derived by the State from liquor license. Hence, for many years yet to come we can not hope for a change, and the removal of the principal cause of insanity, the abolition of the saloon and the pratical extinction of the liquor traffic, and we must, therefore, go on treating results and combating as best we may, the evils resulting from the idiotic policy of licensing men to make widows, orphans, murderers and lunatics.

Seeing that alcoholic insanity is transmitted to succeeding generations, it appears to me that the only rational thing to do is to prevent those succeeding generations; stop the propagation of a race of lunatics. Our four large asylums are always crowded to repletion, with possibly 1000 lunatics in jail awaiting for admittance to the sheltering aegis of the State. Circumstances make it necessary, in order that the most distressing and destitute acute cases may be taken care of, to furlough indefinitely certain of the chronic cases, or to discharge certain incurable but harmless ones, or certain others thought to be "cured" or "improved." These go home and reproduce their kind to fill future asylums yet to be built, and hence the multiplication goes on in geometric progression. Circumstances are such that this furloughing and discharging can not be stopped. The insane increase faster than the State can take care of them. There remains, then, but one resource, and in my deliberate judgment the time has come and the conditions demand that the hopelessly insane males shall be sterilized! Do not be shocked. It is not proposed to castrate them. There is a very simple little operation—painless, void of danger, and which does not destroy the sexual power or the sensations attending its exercise, by which complete sterilization may be effected. And so long as it is made necessary by the stress of circumstances to furlough or discharge a lunatic it is an imperative necessity, in the interest of race integrity and humanity, to sterilize him; and the law should require it.

The operation is "vasectomy." I will describe it presently. A corresponding operation may be performed on women, but it is a graver one and not so sure or so simple. It is by no means original with me, nor is the idea new; we have precedents in certain States where the plan is in operation, notably in Indiana, where such a law has been in operation two years. I quote the law in full, and quote further from an excellent paper by Dr. W. T. Belfield, of Chicago. Dr. Belfield is Secretary of the Chicago Society of Social Hygiene, and this paper, "The Sterilization of Criminals and Other Defectives by Vasectomy," has been published under the auspices of that society.

Dr. Belfield, after setting forth the rapid increase of defectives in his own State, recommends vasectomy, and describes it as follows:

"Sterilization of the male criminal by castration, though often discussed, will probably never secure legal sanction, because it destroys the subject's sexual power; it unsexes a man.

"Vasectomy sterilizes a man without the slightest impairment of his sexual functions; it merely blocks the minute canal (the 'vas') leading from the testis, through which the fertilizing elements of the male must pass to reach the organs which furnish the bulk of the seminal fluid. The absence of these elements from this fluid, though preventing impregnation, causes no impairment of sexual power or pleasure. This is abundantly proven by the robust sexual health of thousands of men who have been unwittingly sterilized through venereal disease, and who never suspect that their procreative functions are not perfectly normal until their marriages prove barren.

"Vasectomy is an office operation; it is painlessly performed in a few minutes under cocaine anesthesia, through a skin cut half an inch long; it entails no wound infection, no confinement to bed; it is less serious than the extraction of a tooth.

"The prevention of procreation by male defectives through vasectomy is not an iridescent dream. In March, 1907, the Indiana Legislature passed a bill authorizing the sterilization of 'confirmed criminals, idiots, imbeciles and rapists' in the State institutions of Indiana; over 800 convicts have been sterilized, some by authority of the State, but over 200 at their own request. This voluntary submission to sterilization by hundreds of convicts, removes the only conceivable opposition to this method of protecting society—the ultra-sentimental.

"In February, 1909, the Oregon Legislature passed a duplicate of the Indiana bill, adding a definition of 'confirmed' criminals.' This term shall be deemed to apply to and include all persons serving a third term in any penitentiary or penal institution upon conviction of a felony.

"While the first, chief and only needed argument for the sterilization of defectives is the protection of society, yet the sentimental may find additional ground for demanding it in the rescue of myriads of criminals, imbeciles and other defectives not yet begotten, from the misery and disaster that must otherwise attend them. Self-interest and altruism, the protection of society and true philanthropy, alike acclaim Indiana's epoch-making advance."

The Indiana sterilization law, enacted in Oregon, also is appended:

"Preamble—Whereas, heredity plays a most important part in the transmission of crime, idiocy and imbecility:

"Therefore, Be it enacted by the General Assembly of the State of Indiana, that on and after the passage of this act it shall be compulsory for each and every institution in the State, entrusted with the care of confirmed criminals, idiots, rapists and imbeciles, to appoint upon its staff, in addition to the regular institutional physician, two skilled surgeons of recognized ability, whose duty it shall be, in conjunction with the chief physician of the institution, to examine the mental and physical condition of such inmates as are recommended by the institutional physician and board of managers. If, in the judgment of this committee of experts and the board of managers, procreation is inadvisable and there is no probability of improvement of the mental condition of the inmate, it shall be lawful for the surgeons to perform such operation for the prevention of procreation as shall be decided safest and most effective. But this operation shall not be performed except in cases that have been pronounced unimprovable."

I make a note here that vasectomy will not do for the rapist or would-be rapist. Nothing but the entire ablation of the genitalia will meet the requirements of his case or be effective.

I agree with Dr. Belfield, who says: "This method of arresting the production of criminals (and lunatics) is, I am bound to believe, one of the coming blessings to humanity," and I earnestly advise that the committee on legislation bring the subject to the attention of our law-makers and ask for the passage of a bill to make it effective in Texas.

